# Effects of aerobic exercises in prediabetes patients: a systematic review and meta-analysis

**DOI:** 10.3389/fendo.2023.1227489

**Published:** 2023-07-13

**Authors:** Yifei Wang, Honglei Li, Dongxue Yang, Mengzhao Wang, Yanbai Han, Hongli Wang

**Affiliations:** ^1^ College of Physical Education and Health, Guangxi Normal University, Guilin, China; ^2^ School of Chemistry and Chemical Engineering, Nanjing University, Nanjing, China

**Keywords:** prediabetes, aerobic exercises, randomized controlled trials, systematic review, meta-analysis

## Abstract

**Aims:**

To evaluate the effects of different durations of continuous aerobic exercise on prediabetic patients.

**Materials and methods:**

The research encompassed randomized controlled trials that examined how various durations of aerobic exercise training affected outcomes related to Body Mass Index (BMI), Fasting blood glucose (FBG), 2-hour plasma glucose (2hPG), and glycated hemoglobin (HbA1c) in individuals diagnosed with prediabetes. PubMed, Embase, Web of Science, and the Cochrane Library were searched as of January 7, 2023. The Cochrane Risk of Bias, version 2 (ROB 2) tool was used to assess the risk of bias.

**Results:**

A total of 10 RCTs with 815 prediabetic patients were included. The average age of the participants was 56.1 years, with a standard deviation of 5.1 years. Among the participants, 39.2% were male. The interventions consisted of aerobic dance, treadmill running, walking, and a combination of aerobic exercises. The training sessions occurred three or four times per week. In prediabetic patients, aerobic exercise demonstrated a significant reduction in BMI compared to the control group, with a weighted mean difference (WMD) of -1.44 kg/m^2^ (95% confidence interval [CI] -1.89, -0.98). There was a decrease in FBG levels, with WMD of -0.51 mmol/L (95% CI -0.70, -0.32). Additionally, aerobic training led to significant improvements in 2hPG levels, with a WMD of -0.76 mmol/L (95% CI -1.14, -0.38). Furthermore, prediabetic patients showed a decrease in HbA1c levels after engaging in aerobic training compared to the control group, with a WMD of -0.34% (95% CI -0.44, -0.23).

**Conclusion:**

In summary, engaging in aerobic exercise can have a significant positive impact on glycemic levels in individuals with prediabetes. It can also lead to reductions in BMI, FBG, 2hPG, HbA1c, and other relevant indicators. The extent of these improvements may vary slightly depending on the duration of the aerobic exercise intervention.

**Systematic review registration:**

PROSPERO https://www.crd.york.ac.uk/PROSPERO/, identifier CRD42023395515.

## 1 Introduction

Diabetes constitutes a significant global health concern in the 21st century, affecting 537 million adults in 2021. This number is projected to increase to 643 million by 2030 and 783 million by 2045 (1). The International Diabetes Federation has predicted that by 2021, diabetes will be responsible for 6.7 million deaths and medical costs exceeding $966 billion, which will pose a substantial social and economic burden on society (1, 2). Most people experience a pre-diabetic phase before developing diabetes (3). Key features of this pre-diabetic phase include impaired fasting glucose (IFG) and impaired glucose tolerance (IGT) (4). Pre-diabetic patients who are obese and physically inactive face a higher risk of developing diabetes compared to others (5). Without timely intervention for pre-diabetics, the risk of developing diabetes is 74% (6). In addition, Kurihara et al. (7) suggest that prediabetes can significantly impact cardiovascular disease and may progress directly to type 2 diabetes (T2D), leading to more severe consequences. Therefore, it is critical to detect and intervene promptly in patients with prediabetes.

According to Hostalek et al. (8), pharmacological treatments such as metformin can be utilized for managing and avoiding diabetes. However, Davidson et al. (9) revealed that metformin is not appropriate for patients with prediabetes. While there are various approaches to treat prediabetes (10), there are still some controversies about interventions for prediabetes. Some studies suggest that lifestyle changes (11) and a healthy diet (12) can reduce the risk of developing diabetes. Furthermore, Kirwan et al. (13) found that appropriate physical activity can reduce the risk of progression to diabetes in patients with prediabetes. Aerobic exercise is widely recognized for its capacity to reduce insulin resistance and mitigate the risk of developing diabetes through aerobic training (14). Kargarfard et al. (15) found that aerobic exercise can have a favorable impact on people with prediabetes. The American Diabetes Association (ADA) recommends at least 150 minutes of moderate-intensity physical activity three times a week in patients with T2D or prediabetes (16). Meanwhile, Hrubeniuk et al. (17) concluded that although exercise may improve glucose tolerance in individuals with prediabetes, the improvement is not considered substantial. This could be attributed to the short duration of the intervention, highlighting the need for further investigation into the optimal prescription of exercise interventions for prediabetic patients.

The objective of this study was to employ meta-analysis to examine the specific effects of aerobic exercise with different training durations on preventing the conversion to T2D in individuals with prediabetes. We conducted an analysis of the distinct alterations in four outcome measures: Body Mass Index (BMI), Fasting blood glucose (FBG), 2-hour plasma glucose (2hPG), and glycated hemoglobin (HbA1c), following various exercise intervention durations. These findings will aid clinicians in designing personalized programs for patients with prediabetes and supporting their recovery.

## 2 Materials and methods

### 2.1 Protocol and registration

The protocol has been registered with the International Registry of Prospective Systematic Reviews (PROSPEROID: CRD42023395515). A systematic literature search was conducted according to PRISMA (Preferred Reporting Items for Systematic Reviews and Meta-Analyses) guidelines (18).

### 2.2 Data sources and searches

Two researchers, W.Y.F. and H.L.L., independently conducted searches in four databases: PubMed, Embase, Web of Science, and the Cochrane Library. A systematic search was performed using the terms “aerobic exercise” AND “prediabetes.” (For a comprehensive list of search terms, please refer to **Supplementary Table S1**). Studies published from the inception of the databases up to January 7, 2023, were included. Additionally, we screened the references of relevant studies to supplement the search results from these databases. No language restrictions were applied in this study.

### 2.3 Eligibility criteria

The inclusion criteria were determined based on PICOS (population, intervention, control, outcome, and study design) criteria (18). The study participants included individuals with prediabetes, including those with IGT and IFG, without any gender or age restrictions. The intervention must be supervised aerobic exercise training. For comparison, the control group should consist of participants without any interventions (referred to as “blank controls”). We included randomized controlled trials (RCTs) that compared aerobic exercise interventions with usual care (no exercise). If the control group implemented a healthy diet, the intervention group also needed to incorporate the same dietary control to ensure that aerobic exercise was the sole intervention. If multiple interventions were available, only data from the aerobic and control groups were extracted. At least one primary outcome was reported (BMI, FBG level, any change in 2hPG or HbA1c).

The criteria for diagnosing prediabetes are as follows: 1) FBG ranging from 100 mg/dL (5.6 mmol/L) to 125 mg/dL (6.9 mmol/L) (IFG). 2) Blood glucose concentration of 140 mg/dL (7.8mmol/L) to 199 mg/dL (11.0 mmol/L) two hours after intake of 75g oral glucose (IGT). 3) HbA1c ranging from 5.7% to 6.4% (4).

Participants who had diabetes, cardiovascular disease, were pregnant, had major musculoskeletal problems, or any other medical conditions that would substantially impact the exercise interventions were excluded from the study. Additionally, individuals who were taking hypoglycemic or lipid-lowering medications were also excluded.

### 2.4 Data extraction

Two investigators independently assessed search results and extracted data using a standardized data extraction format. The following data were extracted: first author, year of publication, study region, study design, age and sex of the included patients, proportions, baseline BMI, type, intensity, frequency, and duration of the aerobic interventions. Any disagreements were resolved through consensus. If a consensus could not be reached, a third reviewer (H.L.W) was consulted.

### 2.5 Assessment of risk of bias

Two reviewers (Y.F.W. and H.L.L) independently rated the risk of bias of the RCTs using the revised Cochrane risk of bias, version 2 (RoB 2) tool. The risk of bias was categorized as high, low, or having some concerns. In cases where there was disagreement between the two researchers, a third party was involved to facilitate discussion and reach a consensus.

### 2.6 Data synthesis and analyses

Stata15.1 software was used for analyzing the continuous outcome indicators of the included studies. The weighted mean difference (WMD) statistic was used to combine effect sizes, and a 95% confidence interval (CI) was provided for each effect size. Effect sizes for the primary outcomes were expressed as WMD. Cochran’s Q and *I^2^* were employed to assess study heterogeneity, with *I^2^* <25% indicating low heterogeneity, 25% to 50% indicating moderate heterogeneity, and > 50% indicating high heterogeneity. The fixed effect model was applied for low and moderate heterogeneity, while the random effect model was used for high heterogeneity. Subgroup analysis was conducted to examine the influence of different time periods on the exercise and its outcomes. To explore the potential publication bias, Egger’s test was employed. The significance level was set at 0.05.

## 3 Results

### 3.1 Study selection

**Figure 1:**
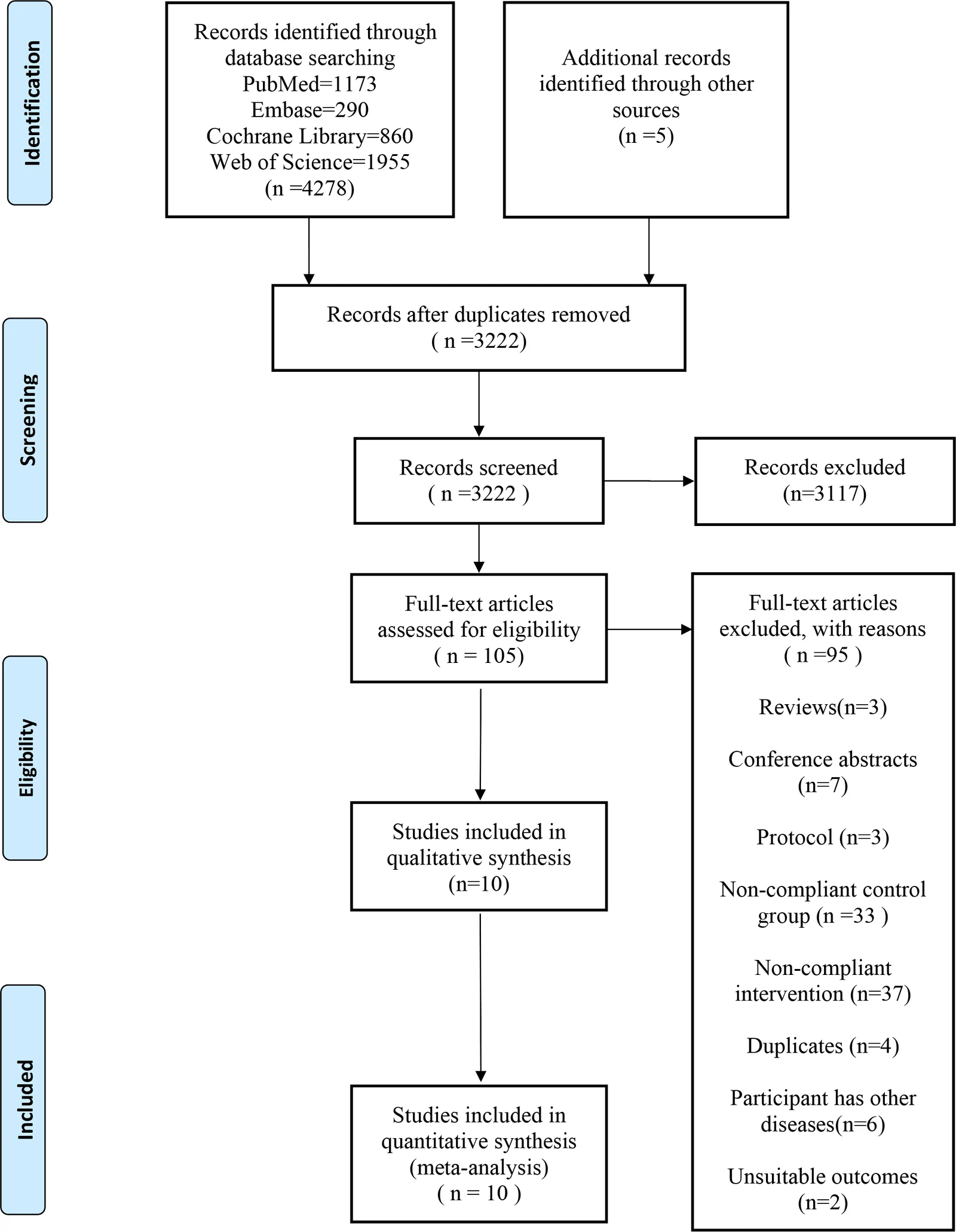
Flowchart of study selection process.

A total of 4283 papers were identified through database searches and other sources. After removing duplicates, 3222 papers remained. Among these, 3117 were excluded based on their titles and abstracts, leaving 105 papers for full-text examination. Finally, 10 RCTs were included in the review and meta-analysis (14, 15, 19–26) (**Figure 1**).

The Cochrane Risk of Bias assessment (ROB2) indicated that eight of the studies had a low risk of bias, while the remaining two studies were found to have some concerns regarding bias (**Supplementary Figure S1**).

### 3.2 Study characteristics

A total of 815 patients with prediabetes were included in the analysis, derived from the 10 articles that were ultimately selected for the study. Among the studies included in the analysis, the average (SD) age of the participants was 56.1 (5.1) years, with males comprising 39.2% of the subjects. The BMI of the participants was provided in nine research, and the type, intensity, frequency, and duration of aerobic exercise were reported in all ten investigations.

All aerobic training was performed under supervised conditions. Modes of training include aerobic dance (14, 19–21, 24, 25), treadmill running (15, 26), walking (15, 22), and mixed aerobic exercise (23). In nine studies, exercise intensity was reported as maximum heart rate (HRmax), maximum heart rate reserve (HRRmax), or heart rate reserve (HRR) with a median intensity and range of 65% (50%-75%). Another study (26) reported on high-intensity interval training (HIIT): 4 x 4-minute intervals at 90% of maximum heart rate (**Supplementary Table S2**).

### 3.3 BMI

**Figure 2:**
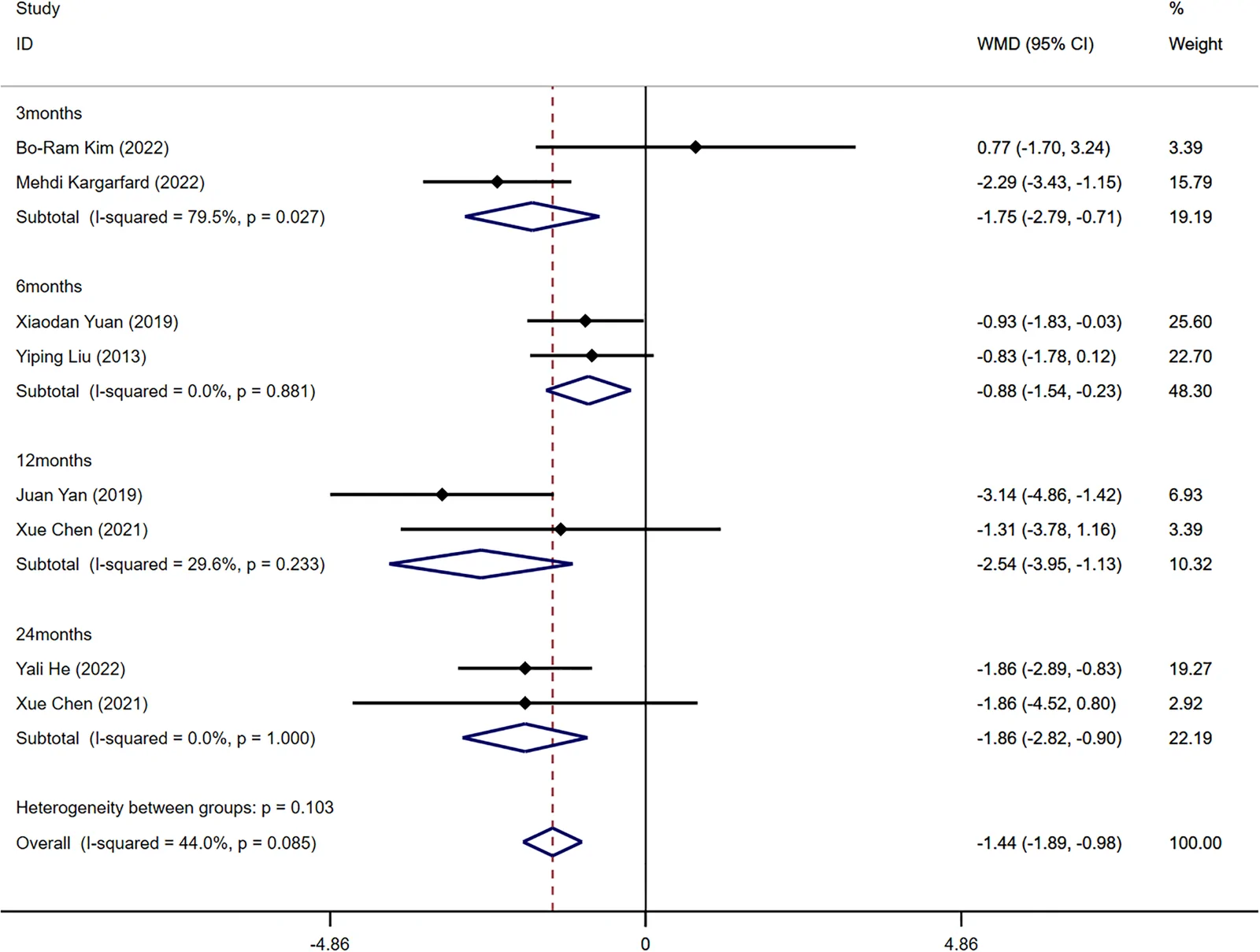
Forest plot of the meta-analysis of the effect of aerobic exercise on BMI.

Seven out of the ten included studies reported BMI indicators (14, 15, 20–23, 25). The combined analysis revealed a significant difference in BMI between the aerobic exercise group and the control group (WMD=-1.44 kg/m^2^, 95%CI -1.89, -0.98, *P* <0.001; heterogeneity: *I^2^ = *44%, *P* = 0.085). To explore the potential impact of exercise at different stages, subgroup analysis was performed based on various training durations. After combining and analyzing data from all four intervention periods (3, 6, 12, and 24 months), significant differences in BMI levels before and after the intervention were identified (3 months: WMD=-1.75 kg/m^2^, 95%CI -2.79, -0.71, *I^2^ = *79.5%, P=0.027; 6 months: WMD=-0.88 kg/m^2^, 95%CI -1.54, -0.23, *I^2^ = *0.0%, *P*=0.881; 12 months: WMD=-2.54 kg/m^2^, 95%CI -3.95, -1.13, *I^2^ = *29.6%, *P*=0.233; 24 months: WMD=-1.86 kg/m^2^, 95%CI -2.82, -0.90, *I^2^ = *0.0%, *P*=1.000) (**Figure 2**). The Egger’s test showed no evidence of publication bias in this result (t=-0.20, *P*=0.850) (**Supplementary Figure S2**).

### 3.4 FBG

**Figure 3:**
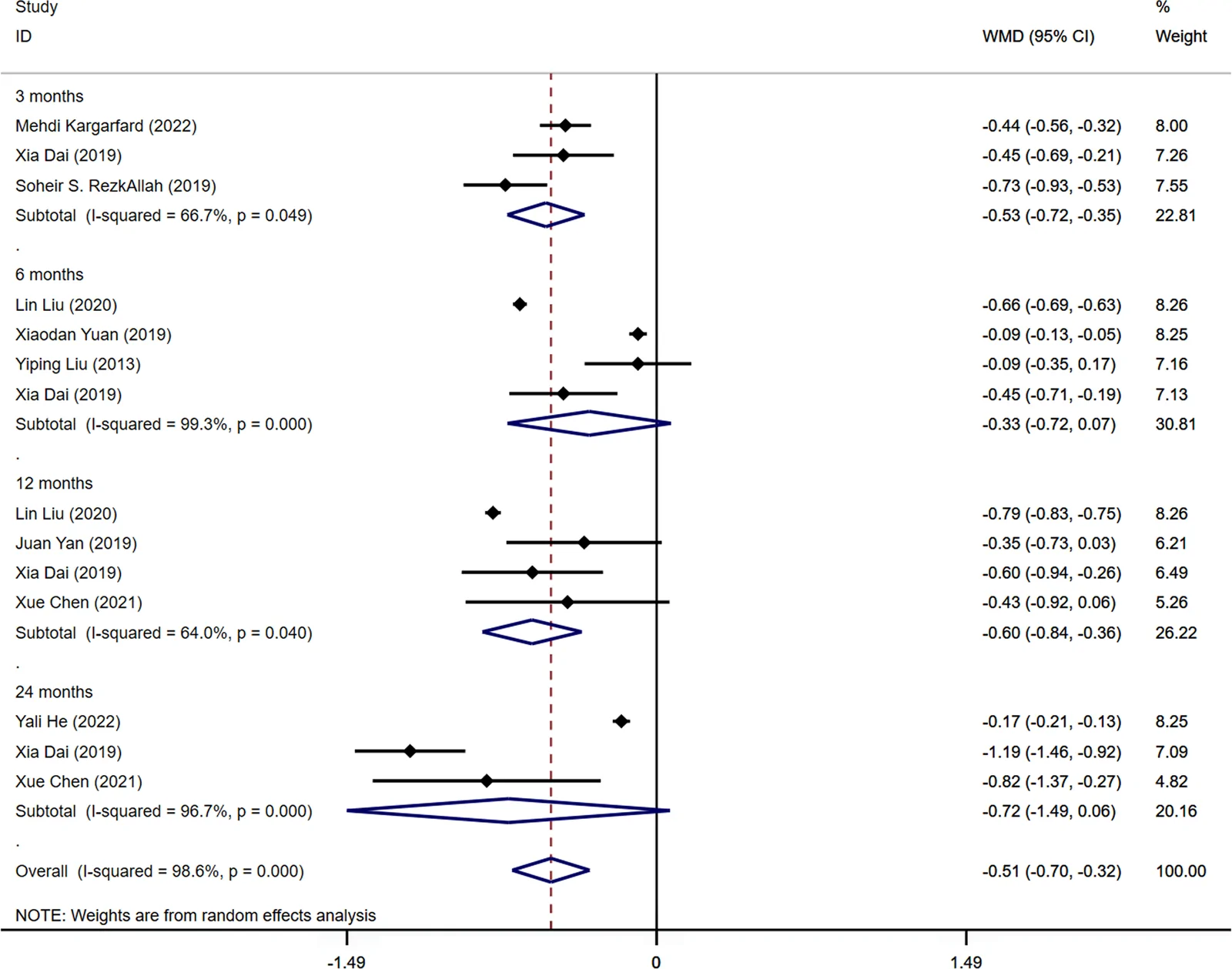
Forest plot of the meta-analysis of the effect of aerobic exercise on FBG.

Nine of the studies included in the analysis provided data on fasting blood glucose (FBG) levels (14, 15, 19–22, 24–26). The pooled results revealed a significant difference in FBG levels between the aerobic exercise group and the control group (WMD=-0.51mmol/L, 95%CI -0.70 -0.32, *I^2^ = *98.6%, *P*<0.001). Significant differences in FBG levels were found during subgroup analysis, specifically when data from three intervention periods (3 and 12 months) were combined and examined (3 months: WMD=-0.53mmol/L, 95% CI -0.72 -0.35, *I^2^ = *66.7%, *P*=0.049; 12 months: WMD=-0.60mmol/L, 95%CI -0.84 -0.36, *I^2^ = *64.0%, *P*=0.040). In contrast, there was no statistically significant difference in FBG between the aerobic exercise group and the control group following 6 and 24 months of intervention (6 months: WMD=-0.33mmol/L, 95%CI -0.72 0.07, *I^2^ = *99.3%, *P*=0.000; 24 months: WMD=-0.72mmol/L, 95%CI -1.49 0.06, *I^2^ = *96.7%, *P*=0.000) (**Figure 3**). The results of the Egger’s test suggested no publication bias in this finding, as indicated by a non-significant test statistic (t=0.01, *P*=0.995). (**Supplementary Figure S3**).

### 3.5 2hPG

**Figure 4:**
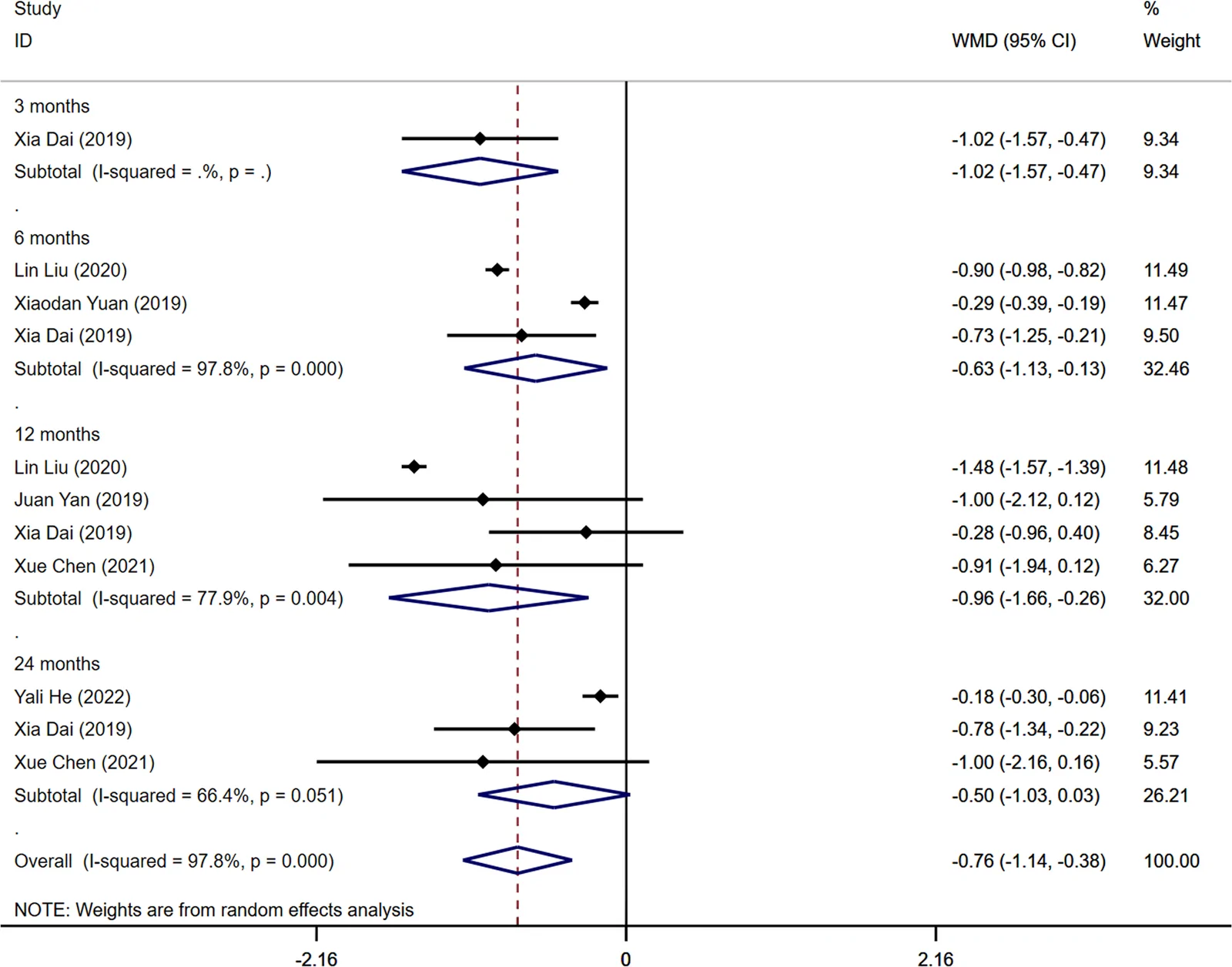
Forest plot of the meta-analysis of the effect of aerobic exercise on 2hPG.

Six studies among those identified in the analysis reported outcome measures of 2-hour postprandial glucose (2hPG) levels (14, 19–21, 24, 25). The combined results demonstrated a significant difference in 2hPG levels between the aerobic exercise group and the control group (WMD=-0.76mmol/L, 95% CI -1.14 -0.38, *I^2^ = *97.8%, *P*<0.001). Statistically significant differences in 2hPG levels were found when analyzing the combined data from all four intervention periods (3, 6, and 12 months), indicating changes in 2hPG levels before and after the intervention (3 months: WMD=-1.02mmol/L, 95%CI -1.57 -0.47; 6 months: WMD=-0.63mmol/L, 95% CI -1.13 -0.13, *I^2^ = *97.8%, *P*=0.000; 12 months: WMD=-0.96mmol/L, 95%CI -1.66 -0.26, *I^2^ = *77.9%, *P*=0.004). Nonetheless, no statistically significant difference was observed in 2hPG levels between the aerobic exercise and control groups after 24 months of intervention (WMD=-0.50mmol/L, 95%CI -1.03 0.03, *I^2^ = *66.4%, *P*=0.051) (**Figure 4**). The Egger’s test indicated no indication of publication bias in this result (t=0.29, *P*=0.779) (**Supplementary Figure S4**).

### 3.6 HbA1c

**Figure 5:**
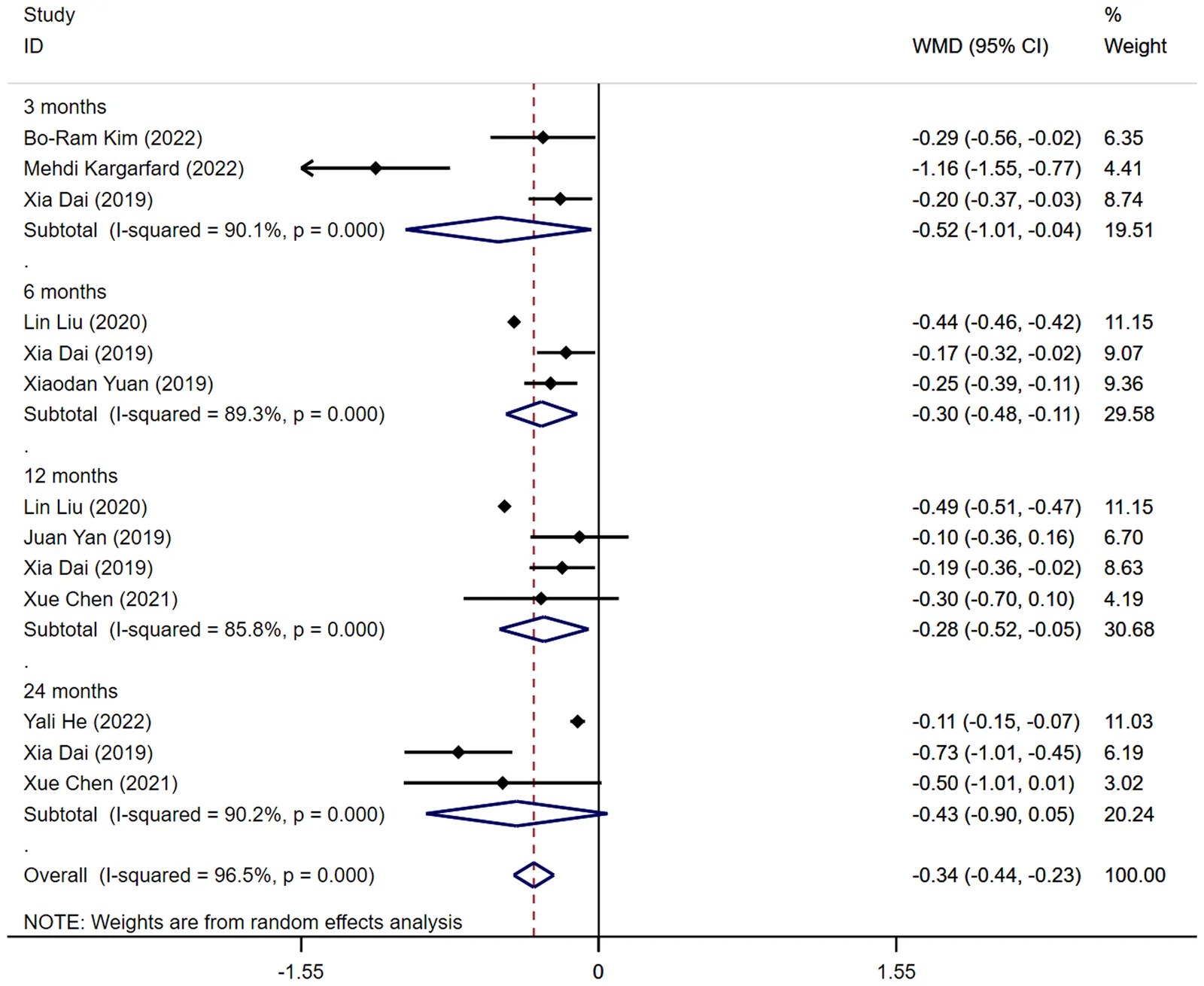
Forest plot of the meta-analysis of the effect of aerobic exercise on HbA1c.

Among the ten studies included in the analysis, seven studies reported outcome measures of HbA1c levels (14, 15, 19, 21, 23–25). The pooled results indicated a statistically significant difference in HbA1c levels between the aerobic exercise group and the control group (WMD=-0.34%, 95CI -0.44 -0.23, *I* ^2^ = 96.5%, *P*<0.001). Significant statistical differences in HbA1c levels were detected when analyzing the combined data from all four intervention periods (3, 6, and 12 months) and comparing the measurements before and after the intervention (3 months: WMD=-0.52%, 95%CI -1.01 -0.04, *I* ^2^ = 90.1%, *P*=0.000; 6months: WMD=-0.30%, 95%CI -0.48 -0.11, *I* ^2^ = 89.3%, *P*<0.001; 12 months: WMD=-0.28%, 95%CI -0.52 -0.05, *I* ^2^ = 85.8%, *P*=0.000). After 24 months of intervention, no statistically significant difference was found in HbA1c levels between the aerobic exercise group and the control group (WMD=-0.43%, 95%CI -0.90 0.05, *I^2^ = *90.2%, *P*=0.000) (**Figure 5**). The result was not influenced by publication bias, as indicated by Egger’s test (t=0.90, *P*=0.388) (**Supplementary Figure S5**).

### 3.7 Sensitivity analyses

Sensitivity analysis was conducted on a case-by-case basis, and the results remained stable and reliable after the analysis (**Supplementary Figures S6**-**S9**).

## 4 Discussion

This systematic review and meta-analysis revealed that aerobic exercise training had positive effects on reducing BMI, FBG, 2hPG, and HbA1c levels in individuals with prediabetes. Subgroup analysis demonstrated varying improvements in these indicators based on different training durations among patients with prediabetes. Collectively, these findings suggest that aerobic exercise can be utilized to enhance outcomes and reduce the risk of developing diabetes in individuals exhibiting prediabetes symptoms.

This study revealed a reduction in BMI (WMD -1.44 kg/m^2^) among patients with prediabetes who underwent aerobic exercise. The intervention demonstrated effectiveness and significance, as indicated by the precise estimation of WMD (95% CI). However, the findings pertaining to this outcome indicator were inconsistent with the results reported by Huang et al. (27). This could be attributed to differences in the study population, intervention length, intensity, frequency, and type of aerobic exercise intervention. Nevertheless, since the studies in our meta-analysis were similar in terms of training intensity and frequency, as well as exercise mode, the resulting effects would be more probable. Based on our subgroup analysis, we found that aerobic exercise was most effective in reducing BMI in individuals with prediabetes after 12 months of training (WMD -2.54kg/m^2^). This could be due to the fact that exercise in combination with a personalized nutritional diet has been shown to be more effective in weight control than exercise alone, as suggested by Yan (21) and confirmed by Magkos et al. (5). Magkos et al. (5) found that as the BMI of prediabetic patients with overweight or obesity increased, their risk of developing T2D also increased. The combination of an exercise intervention along with a diet that restricts calorie intake has been found to improve symptoms in around 80% of obese and T2D patients. This approach can result in weight loss and better glycemic control (5). Unfortunately, because of the small number of included literature, more literature is still needed to demonstrate this. A previous prospective study by Eriksson et al. (28), found that body mass index and weight loss after exercise intervention improved the metabolic status of overweight or obese people with prediabetes, normalized glucose tolerance in more than 50% of IGT patients, and improved glucose tolerance. which was associated with weight loss, promoting recovery from prediabetes and preventing the development of diabetes (29, 30).

Collectively, FBG improved after aerobic exercise intervention (WMD -0.51mmol/L). This finding is consistent with previous studies. The precision of the estimated WMD (95% CI) indicates the effectiveness of the intervention. Although our subgroup analysis did not show a significant effect of aerobic training after 6 months and no statistically significant difference between the intervention and control groups, we believe that the possible reason for this may be the large age gap between one of the included studies and the other studies, as well as the fact that the population consisted only of IGT patients (22).

Aerobic training had a significant effect in reducing 2hPG levels in individuals with prediabetes when compared to the control group, as indicated by a WMD of -0.76 mmol/L. The precision of the estimated WMD (95% CI) further supported the beneficial impact of the intervention, with the upper limit suggesting a higher effect. Our subgroup analysis showed significant changes in 2hPG at training up to 3, 6, and 12 months. At one year of intervention, the study conducted by Liu et al. (19) reported the most significant improvement in the 2hPG index, with a WMD of -1.48 mmol/L and a 95%CI of (-1.57, -1.39). This finding can be attributed to the effectiveness of combining exercise and a healthy diet, which was shown to be more effective than exercise alone. Furthermore, AT was found to be more effective than resistance training in improving 2hPG levels in patients with IGT, a finding that is also supported by the study conducted by Malin et al. (31). The meta-analysis conducted by Hrubeniuk et al. (17) concluded that exercise improves glucose tolerance in prediabetic patients, particularly when the intervention includes both aerobic training and resistance training. In contrast, our study is the first meta-analysis to demonstrate that aerobic exercise training alone effectively improves 2hPG levels in prediabetic patients.

Exercise reduced HbA1c in prediabetic patients (WMD -0.34%) and judging by the precision of the estimated WMD (95% CI), the intervention was effective, although the effect size was not very high. According to a meta-analysis conducted by Yang et al. (32), aerobic exercise was found to be more effective than resistance exercise in reducing HbA1c levels in diabetic patients following exercise intervention. In the future, it may be worth considering combined resistance training to assess the extent of improvement in HbA1c levels in prediabetic patients.

Our meta-analysis demonstrates a clear correlation between the duration of aerobic training intervention and the improvement in FBG, 2hPG, HbA1c, and BMI indexes among prediabetic patients. The most notable effects of exercise on each index were observed when the intervention duration was extended to one year. Despite the improvement, the three outcome indicators of FBG, 2hPG, and HbA1c were no longer statistically different between the two groups after 24 months of the exercise intervention. We speculate that the possible reasons for this are the average age of the participants included at this training node is 60 years, and the included population has inconsistent characteristics, probably due to some research incorporating a population of obese patients. Existing research indicates that even as the duration of exercise intervention increases, a small percentage of prediabetic patients fail to achieve the predicted results after two years, exhibit poor response to exercise, and eventually develop diabetes (14). Furthermore, the effects of increased insulin resistance with age, impaired islet function, and impaired insulin secretion may also contribute to the increased incidence of diabetes (33), providing further explanation for our subgroup analysis. Another possible reason is that some of the participants had a family history of diabetes and already reached the threshold after the first year of training, after which secondary diabetes may be influenced by genetic factor (34).

### 4.1 Strengths and limitations of this study

Our systematic review and meta-analysis provide an overview of the effectiveness of aerobic exercise therapy in patients with prediabetes. Although the studies included in this research have some limitations, the low-risk diversity displayed in the outcomes implies that the findings are reliable. In the included studies, the type of aerobic exercise chosen was suitable for the general population, promoting long-term adherence and providing additional benefits. Seven of the studies had training intensities ranging from 60% to 70%, and the training was supervised by professionally trained personnel, ensuring high compliance. Aerobic dance was the most frequently used exercise modality and gained high popularity among elderly Chinese women. In recent years, exercise therapy has been extensively demonstrated as a cost-effective, user-friendly, safe, and devoid-of-side-effects intervention for preventing or treating diabetes, distinguishing it from other approaches (35). Additionally, our study encompassed more outcome indicators in comparison to the meta-analysis conducted by Hrubeniuk et al. (17). A subgroup analysis was conducted, capturing the effects of various intervention durations on outcome indicators. We also included a larger number of subjects compared to the meta-analysis conducted by Huang et al. (27) to comprehensively assess the true impact of different durations of continuous aerobic exercise on patients with prediabetes. Yang (32) et al.’s meta-analysis observed that aerobic training was more effective in decreasing BMI in diabetic patients than resistance training. This finding provides valuable insights that can be applied to designing training programs for prediabetic patients. In the future, we also aim to explore the effect of combined aerobic and resistance training on prediabetic patients.

While our findings indicate that aerobic exercise training may lead to significant improvements in indicators among individuals with prediabetes, the results are subject to some degree of uncertainty due to the presence of heterogeneity, inaccuracies, inconsistencies, and the inability to implement blinded exercise interventions across various studies. In the conducted meta-analysis, aerobic exercise interventions were grouped together. It is important to acknowledge that the observed heterogeneity across the studies may be attributed to variations in the nature, duration, and intensity of the exercise programs. Our research did not differentiate specifically between patients with IGT and IFG. The number of primary literature sources included in our study was limited, and the conclusions drawn from the meta-analysis may be somewhat questionable. In the future, more high-quality studies are needed to obtain more precise results. Additionally, certain indicators such as HS-CRP, which are associated with the incidence of diabetes, could not be included in the meta-analysis due to a lack of available primary studies for analysis. Our study only considered aerobic training studies and excluded those that incorporated resistance training in the control group. However, Grøntved et al. (36) conducted a prospective cohort study that revealed a reduction of approximately 35% and 50% in the risk for men who performed resistance training or aerobic exercise for at least 150 minutes per week, respectively. Incorporating resistance training with aerobic exercise seems to be beneficial in averting T2D. For better generalization, it is recommended to select exercises that have a broader appeal when incorporating resistance training.

### 4.2 Implications for clinicians

FBG, 2hPG, and HbA1c are three distinct indicators related to glucose levels, each reflecting different time periods and factors that influence them. The correlation between these indicators can lead to similarities or differences in the analysis results. Therefore, when assessing long-term glycemic control in diabetic patients, it is essential to measure these indicators simultaneously to obtain more comprehensive and accurate information. Despite the limitations of the studies included, this systematic review and meta-analysis offer an informative overview of the effectiveness of aerobic exercise training at various durations in patients with prediabetes. This summary can serve as a valuable resource for clinical discussions and decision-making processes.

For patients with prediabetes, BMI and HbA1c are crucial indicators in assessing the effects of exercise. It is essential for healthcare professionals to determine the ideal BMI range for older adults, considering that overweight and obese patients may require significant weight loss to decrease their risk of developing diabetes and reverse prediabetes symptoms. Previous prospective cohort studies have suggested that lifestyle modifications, such as exercise and dietary interventions, can effectively prevent and lower the occurrence of T2D in individuals with prediabetes. Furthermore, interventions solely focused on aerobic exercise have been found to reduce the risk of T2D by 46% (37). Based on the findings of this study, exercise training for approximately one year may be recommended for prediabetic patients. However, if there is no significant improvement in the indicators after one year, healthcare professionals should consider additional interventions, such as medication or other treatments.

Our study included middle-aged and elderly participants with a mean age of 56.1 years. Considering the rising prevalence of diabetes in younger populations, future research should explore the impact of exercise interventions on children and adolescents with diabetes and prediabetes (38). This necessitates additional high-quality RCTs to evaluate the impact of various training durations on diabetes incidence, determine the optimal exercise intensity, duration, and frequency, explore the potential benefits of combining aerobic and resistance training, and compare the advantages of different training types for individuals with prediabetes. Implementing exercise interventions for prediabetic patients to improve their indicators and prevent the progression of diabetes can significantly alleviate the global burden of this disease on public health.

## 5 Conclusion

In conclusion, our systematic review and meta-analysis have showcased the positive effects of aerobic exercise training alone on individuals with prediabetes. We observed significant reductions in various indicators when the training was continued for approximately one year. These findings provide valuable guidance for healthcare professionals and patients alike to follow.

## Data availability statement

The original contributions presented in the study are included in the article/**Supplementary Material**. Further inquiries can be directed to the corresponding author.

## Author contributions

YW, HW, and YH conceived and designed the study. YW and HL conducted the scientific literature search. YW and HL performed the literature screening, extracted data, and appraised the quality of the included studies. YW, DY, and MW carried out the statistical analyses. YW and HW wrote the first draft of the paper. YH and HW supervised the work. All authors contributed to the article and approved the submitted version.
