# Correction: Effects of aerobic exercises in prediabetes patients: a systematic review and meta-analysis

**DOI:** 10.3389/fendo.2026.1966280

**Published:** 2026-10-14

**Authors:** Yifei Wang, Honglei Li, Dongxue Yang, Mengzhao Wang, Yanbai Han, Hongli Wang

**Affiliations:** 1 College of Physical Education and Health, Guangxi Normal University, Guilin, China; 2 School of Chemistry and Chemical Engineering, Nanjing University, Nanjing, China

**Keywords:** prediabetes, aerobic exercises, randomized controlled trials, systematic review, meta-analysis

In the published article, there was an error in **Figure 5** as published. Identical results for HbA1c at 6 months and 12 months were reported in **Figure 5**. The corrected **Figure 5** appear below.

**Figure 5:**
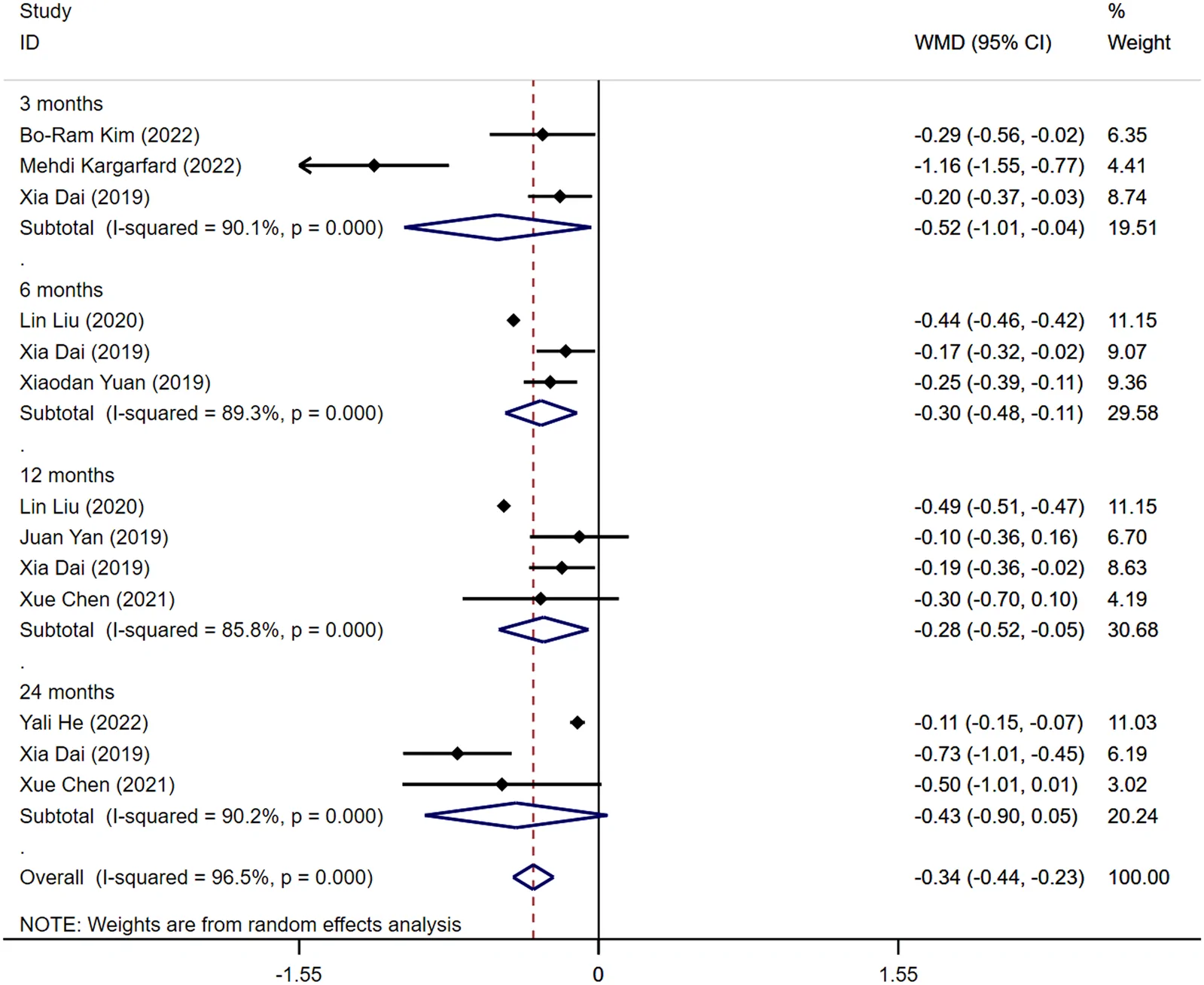
Forest plot of the meta-analysis of the effect of aerobic exercise on HbA1c.

In the published article, there was an error. Identical results for HbA1c at 6 months and 12 months were reported. A correction has been made to the **Results** section of the **Abstract** and to Section 3.6, where the same error appeared.

This sentence previously stated:

“Furthermore, prediabetic patients showed a decrease in HbA1c levels after engaging in aerobic training compared to the control group, with a WMD of -0.34% (95% CI -0.45, -0.23)”.

The corrected sentence appears below:

“Furthermore, prediabetic patients showed a decrease in HbA1c levels after engaging in aerobic training compared to the control group, with a WMD of -0.34% (95% CI -0.44, -0.23).”

This sentence previously stated:

“The pooled results indicated a statistically significant difference in HbA1c levels between the aerobic exercise group and the control group (WMD=-0.34%, 95CI -0.45 -0.23, *I^2^* = 96.5%, *P* < 0.001)”.

The corrected sentence appears below:

“The pooled results indicated a statistically significant difference in HbA1c levels between the aerobic exercise group and the control group (WMD=-0.34%, 95CI -0.44 -0.23, *I^2^* = 96.5%, *P* < 0.001)”

This sentences previously stated:

“6 months: WMD=-0.28%, 95%CI -0.52 -0.05, *I^2^* = 85.8%, *P* < 0.001” “Egger (t=1.00, *P* = 0.337)”

The corrected sentences appear below:

“6 months: WMD=-0.30%, 95%CI -0.48 -0.11, *I^2^* = 89.3%, *P* < 0.001” “Egger (t=0.90, *P* = 0.388)”

In the published article, the *P* values for the pooled effect and for heterogeneity of BMI were not clearly distinguished. A correction has been made to Section 3.3.

This sentence previously stated:

“The combined analysis revealed a significant difference in BMI between the aerobic exercise group and the control group (WMD=-1.44 kg/m², 95% CI -1.89, -0.98, *I²* = 44%, *P* < 0.001).”

The corrected sentence appears below:

“The combined analysis revealed a significant difference in BMI between the aerobic exercise group and the control group (WMD = –1.44 kg/m², 95% CI -1.89, -0.98, *P* < 0.001; heterogeneity: *I²* = 44%, *P* = 0.085).”

In the published article, there was an error in **Supplementary Figure S5**, **S9**. The Egger’s test and sensitivity analysis results in these figures have been revised based on corrected HbA1c data. The supplementary material has been updated.

The original article has been updated.

