# Supplementary material for: Effects of aerobic exercises in prediabetes patients: a systematic review and meta-analysis
Source: Front Endocrinol (Lausanne). 2023 Jul 13;14:1227489. doi: 10.3389/fendo.2023.1227489 (PMC10374027; doi:10.3389/fendo.2023.1227489)
Supplement: Supplementary file 1 [file DataSheet_1.docx]

Supplementary Material

Effects of aerobic exercises in prediabetes patients: a systematic review and meta-analysis

Yifei Wang, Honglei Li, Dongxue Yang, Mengzhao Wang, Yanbai Han, Hongli Wang*

*** Correspondence:** Hongli Wang: whongli2004@gxnu.edu.cn

# Supplementary Figures and Tables

## Supplementary Figures


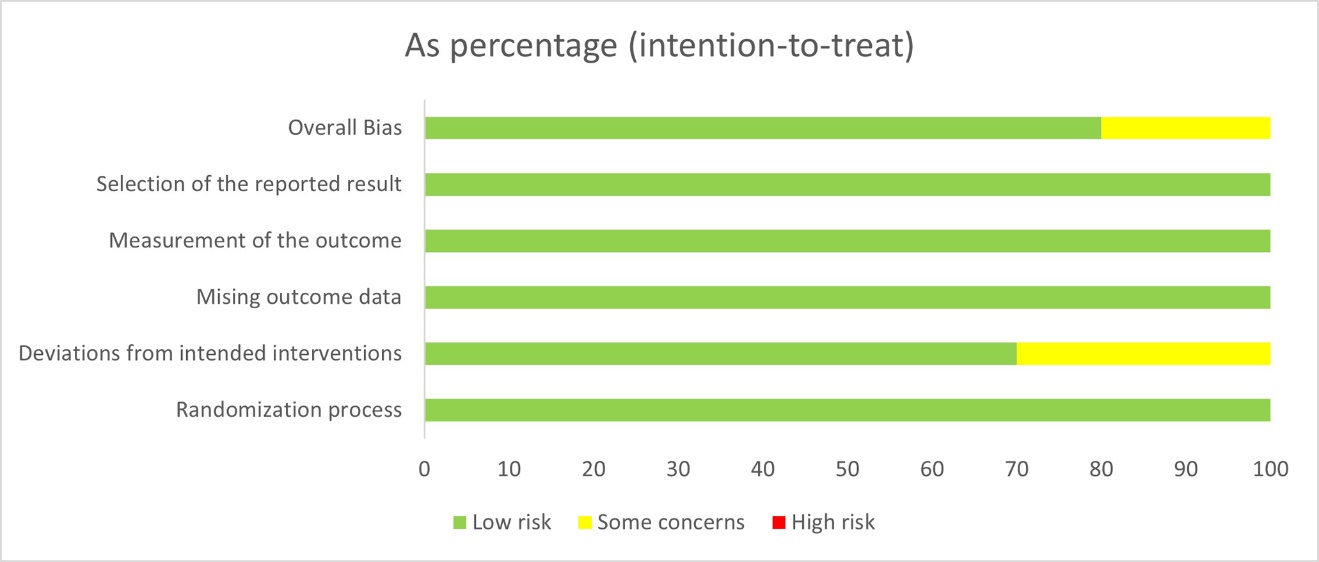


**Supplementary Figure S1.** Risk of bias assessment of included studies.

**
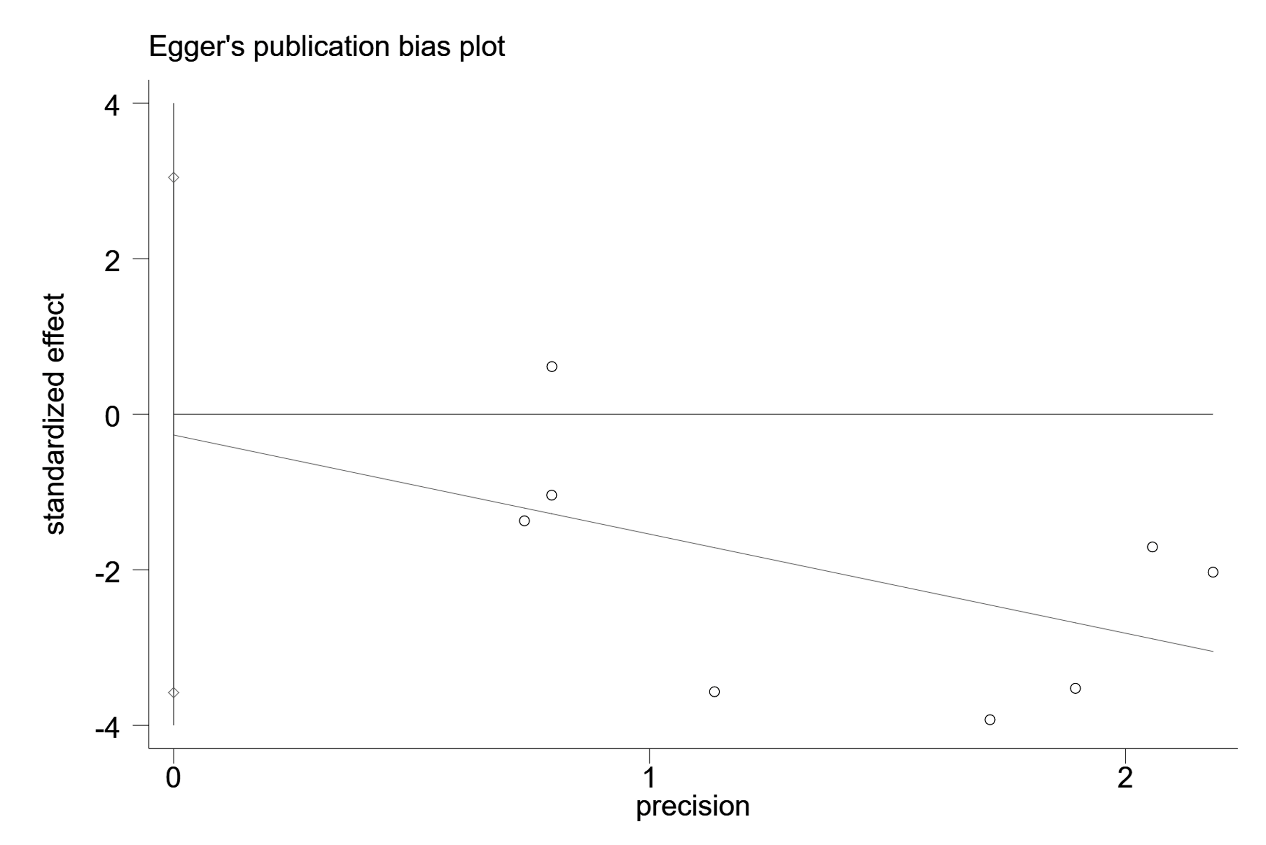
**

**Supplementary Figure S2.** The Egger's funnel diagram of BMI.

**
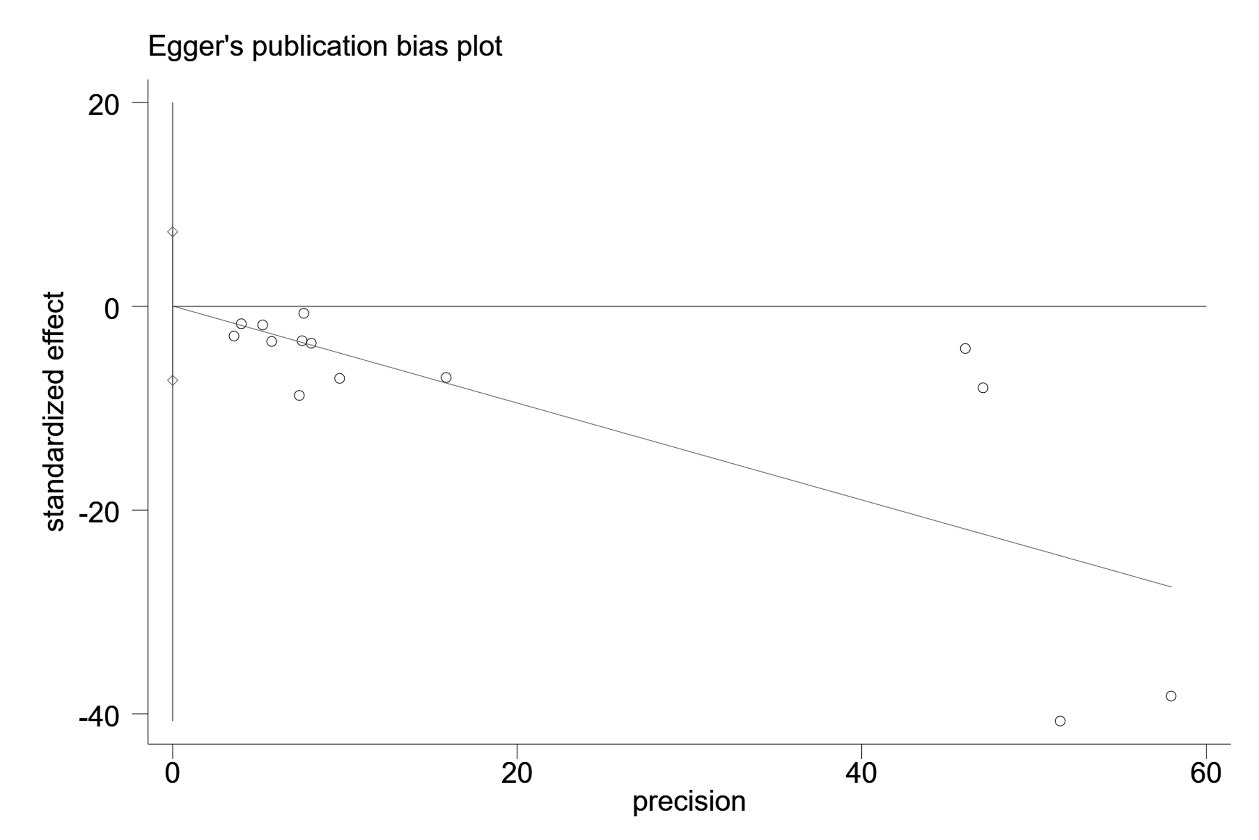
**

**Supplementary Figure S3.** The Egger's funnel diagram of FBG.

**
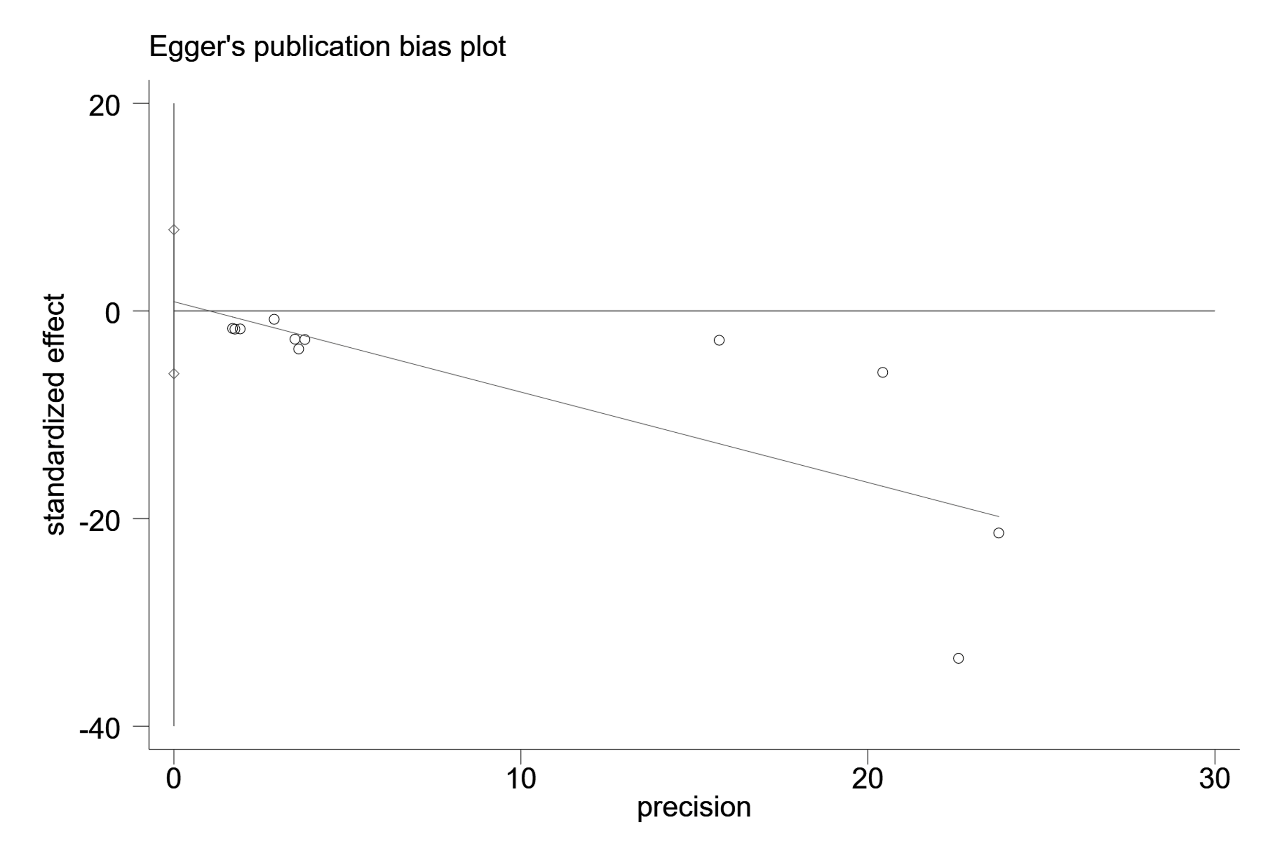
**

**Supplementary Figure S4.** The Egger's funnel diagram of 2hPG.

**
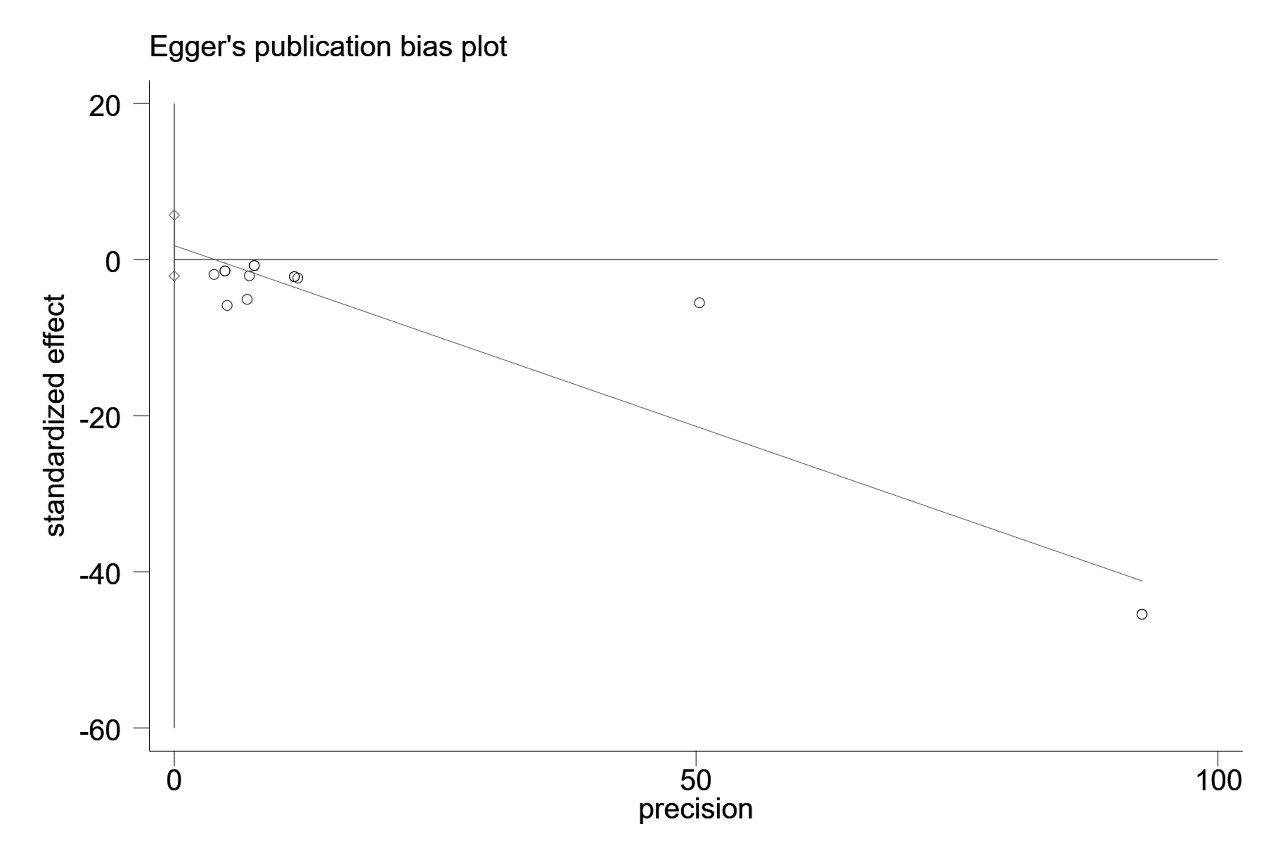
**

**Supplementary Figure S5.** The Egger's funnel diagram of HBA1c.

**
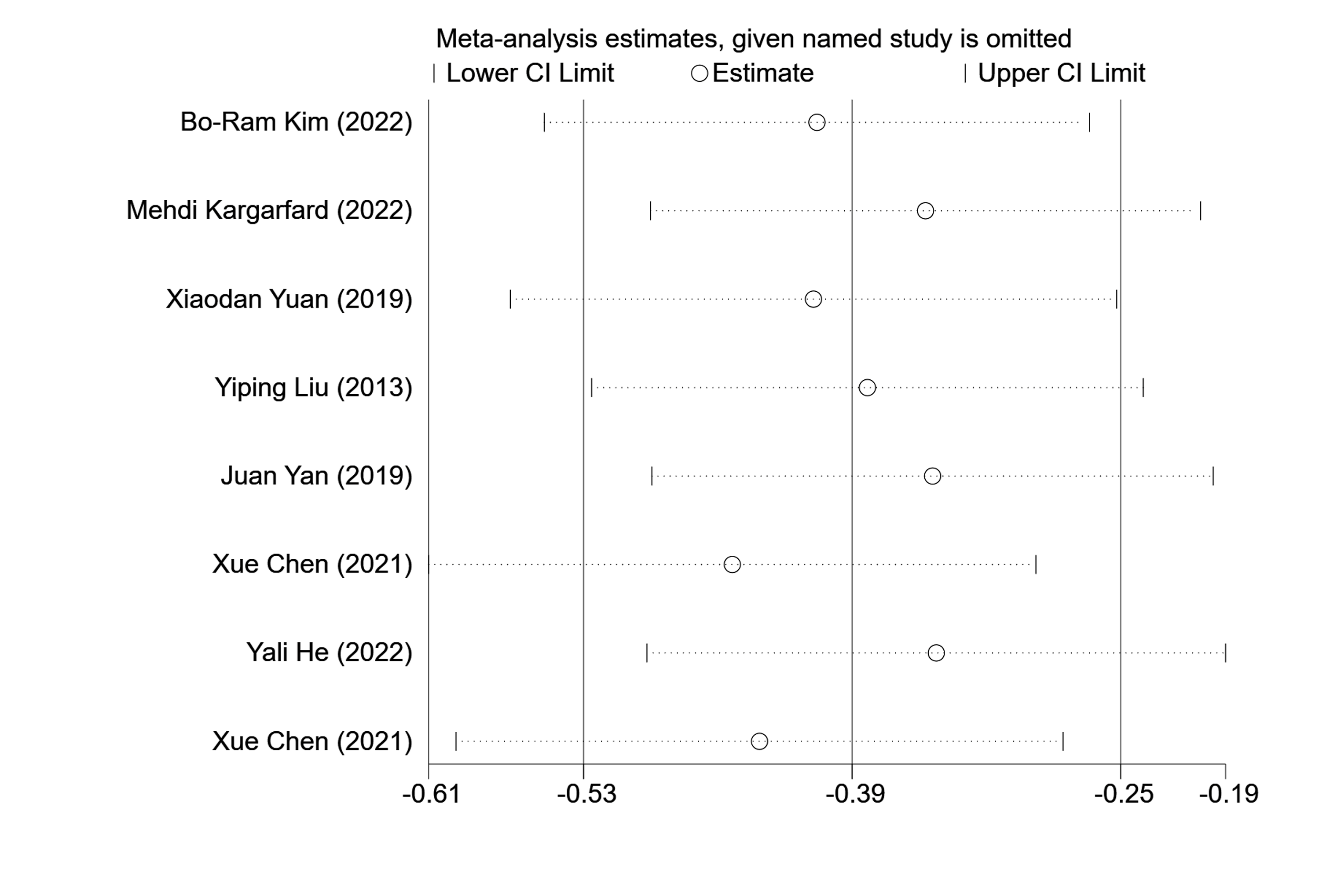
 Supplementary Figure S6.** The Sensitivity Analysis of BMI.

**
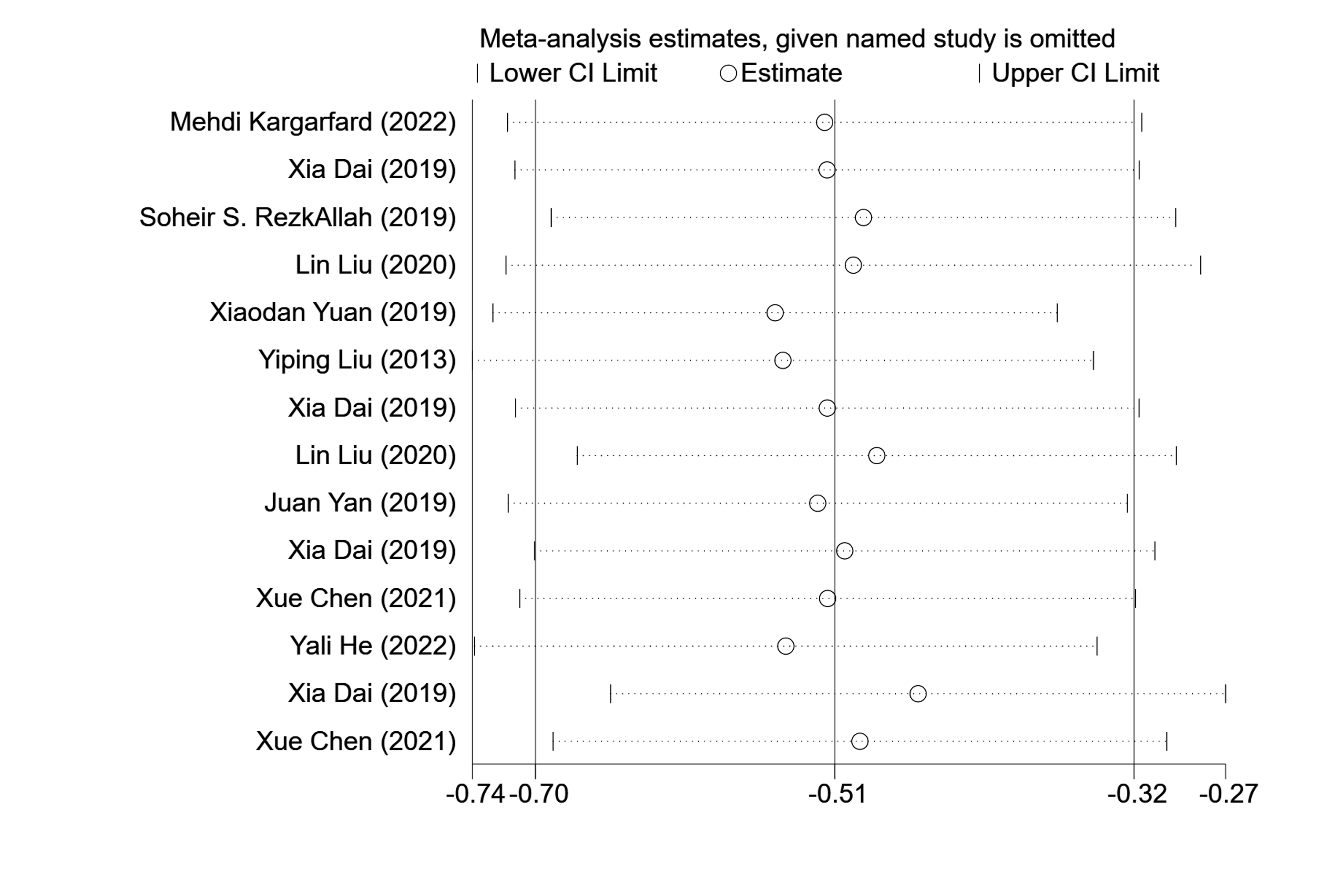
**

**Supplementary Figure S7.** The Sensitivity Analysis of FBG.

**
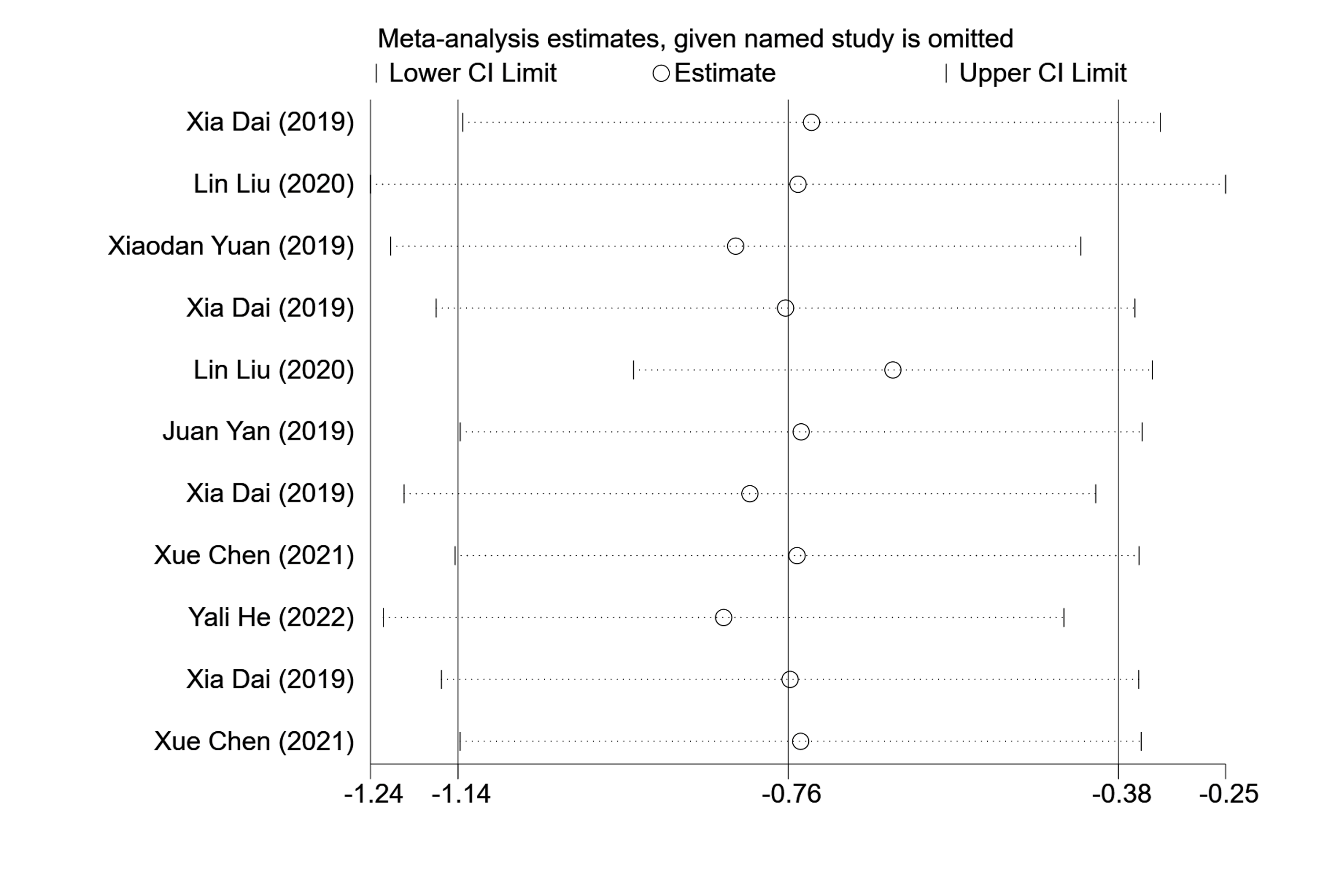
**

**Supplementary Figure S8.** The Sensitivity Analysis of 2hPG.

**
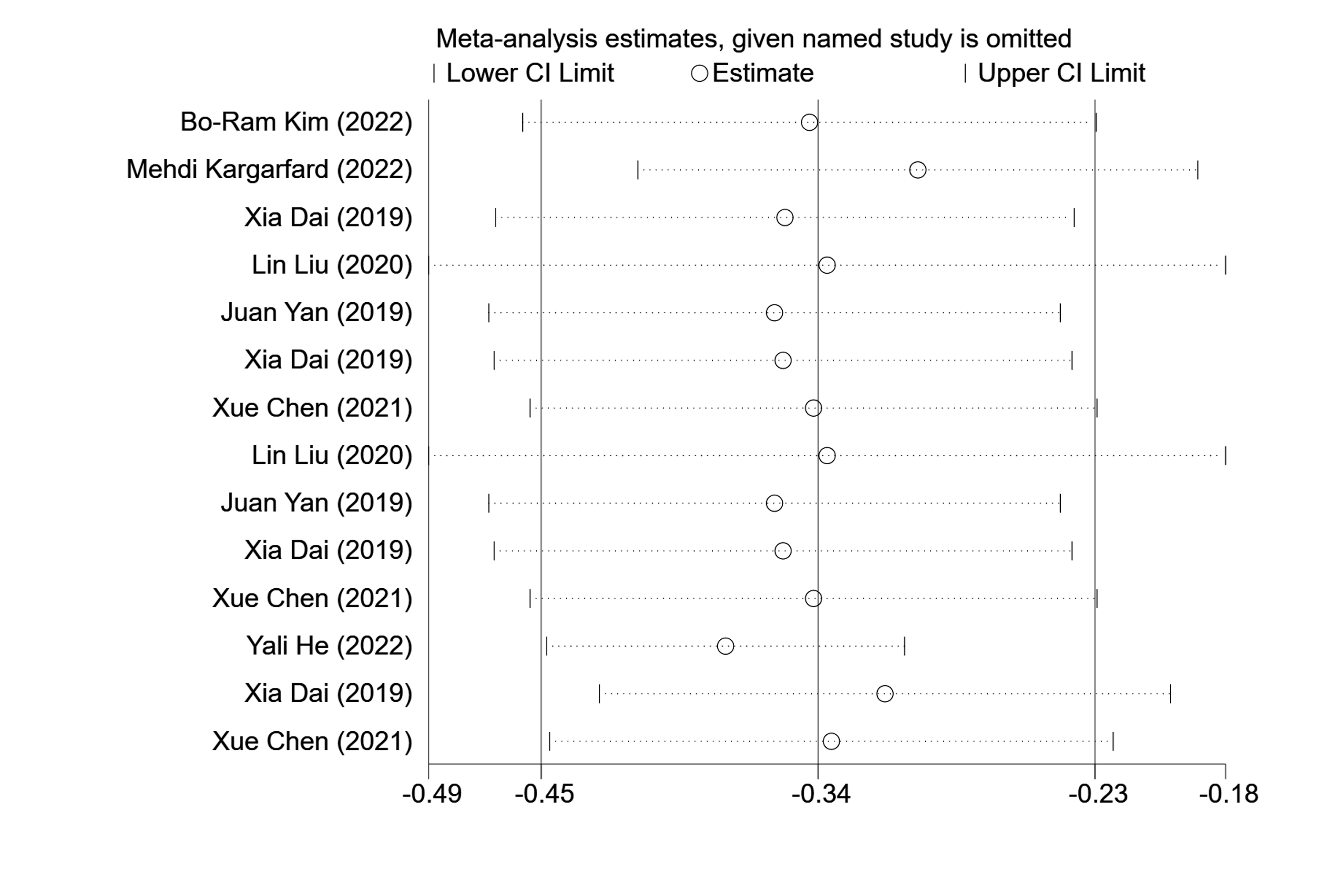
**

**Supplementary Figure S9.** The Sensitivity Analysis of HbA1c.

## Supplementary Tables

**Supplementary Table S1**. Search strategy.

Cochrane-search-history

| ID | Search |
| --- | --- |
| #1 | MeSH descriptor: [Prediabetic State] explode all trees |
| #2 | Prediabetic States |
| #3 | State, Prediabetic |
| #4 | States, Prediabetic |
| #5 | Prediabetes |
| #6 | #1 OR #2 OR #3 OR #4 OR #5 |
| #7 | MeSH descriptor: [Exercise] explode all trees |
| #8 | Exercises, Acute |
| #9 | Acute Exercises |
| #10 | Exercise, Acute |
| #11 | Acute Exercise |
| #12 | Training, Exercise |
| #13 | Trainings, Exercise |
| #14 | Exercise Trainings |
| #15 | Exercise Training |
| #16 | Exercises, Isometric |
| #17 | Isometric Exercises |
| #18 | Isometric Exercise |
| #19 | Exercise, Isometric |
| #20 | Exercise, Aerobic |
| #21 | Exercises, Aerobic |
| #22 | Aerobic Exercise |
| #23 | Aerobic Exercises |
| #24 | Physical Exercises |
| #25 | Exercises |
| #26 | Physical Exercise |
| #27 | Physical Activity |
| #28 | Activity, Physical |
| #29 | Activities, Physical |
| #30 | Exercise, Physical |
| #31 | Exercises, Physical |
| #32 | Physical Activities |
| #33 | #7 OR #8 OR #9 OR #10 OR #11 OR #12 OR #13 OR #14 OR #15 OR #16 OR #17 OR #18 OR #19 OR #20 OR #21 OR #22 OR #23 OR #24 OR #25 OR #26 OR #27 OR #28 OR #29 OR #30 OR #31 OR #32 |
| #34 | #6 AND #33 |

Search Name: Cochrane-search-history

Last Saved: 13/01/2023 13:38:45

1. Embase-search-history

| No. | Query | Results |
| --- | --- | --- |
| #7 | #3 AND #6 | 290 |
| #6 | #4 OR #5 | 22573 |
| #5 | 'aerobic dance'/exp OR 'aerobic dance' OR (aerobic AND ('dance'/exp OR dance)) OR 'aerobic dancing':ab,ti OR aerobics:ab,ti OR 'aerobics exercise':ab,ti OR 'dancing, aerobic':ab,ti OR 'exercise, aerobic':ab,ti OR 'low impact aerobic exercise':ab,ti OR 'low impact aerobics':ab,ti OR 'step aerobics':ab,ti | 22573 |
| #4 | 'aerobic exercise'/exp | 21362 |
| #3 | #1 OR #2 | 74174 |
| #2 | chemical diabetes'/exp OR 'chemical diabetes' OR (chemical AND ('diabetes'/exp OR diabetes)) OR 'chemical diabetes mellitus':ab,ti OR 'diabetes mellitus, potential':ab,ti OR 'diabetes, chemical':ab,ti OR 'diabetes, latent':ab,ti OR 'genetic prediabetes':ab,ti OR 'glucose tolerance impairment':ab,ti OR 'glucose tolerance, potentially impaired':ab,ti OR 'impaired glucose tolerance, potential':ab,ti OR 'latent diabetes':ab,ti OR 'latent diabetes mellitus':ab,ti OR 'potential diabetes':ab,ti OR 'potential diabetes mellitus':ab,ti OR 'potential glucose tolerance impairment':ab,ti OR 'pre diabetes mellitus':ab,ti OR prediabetes:ab,ti OR 'prediabetes mellitus':ab,ti OR 'prediabetic stage':ab,ti OR 'prediabetic state':ab,ti | 74174 |
| #1 | 'impaired glucose tolerance'/exp | 36356 |

Date: 2023/1/7

1. PubMed-Search-History

| Search number | Query | Results |
| --- | --- | --- |
| 5 | (#1 OR #2) AND (#3 OR #4) | 1,173 |
| 4 | Exercise[MeSH Terms] | 239,850 |
| 3 | "Exercises"[Title/Abstract] OR "Physical Activity"[Title/Abstract] OR "Activities, Physical"[Title/Abstract] OR "Activity, Physical"[Title/Abstract] OR "Physical Activities"[Title/Abstract] OR "Exercise, Physical"[Title/Abstract] OR "Exercises, Physical"[Title/Abstract] OR "Physical Exercise"[Title/Abstract] OR "Physical Exercises"[Title/Abstract] OR "Acute Exercise"[Title/Abstract] OR "Acute Exercises"[Title/Abstract] OR "Exercise, Acute"[Title/Abstract] OR "Exercises, Acute"[Title/Abstract] OR "Exercise, Isometric"[Title/Abstract] OR "Exercises, Isometric"[Title/Abstract] OR "Isometric Exercises"[Title/Abstract] OR "Isometric Exercise"[Title/Abstract] OR "Exercise, Aerobic"[Title/Abstract] OR "Aerobic Exercise"[Title/Abstract] OR "Aerobic Exercises"[Title/Abstract] OR "Exercises, Aerobic"[Title/Abstract] OR "Exercise Training"[Title/Abstract] OR "Exercise Trainings"[Title/Abstract] OR "Training, Exercise"[Title/Abstract] OR "Trainings, Exercise"[Title/Abstract] | 231,288 |
| 2 | Prediabetic State[MeSH Terms] | 8,755 |
| 1 | "Prediabetic States"[Title/Abstract] OR "State, Prediabetic"[Title/Abstract] OR "States, Prediabetic"[Title/Abstract] OR "Prediabetes"[Title/Abstract] | 9,839 |

1. WOS-search-history

| # | Search | Results |
| --- | --- | --- |
| 1 | TS=(Prediabetic State) OR TS=(Prediabetic States) OR TS=(State, Prediabetic) OR TS=(States, Prediabetic) OR TS=(Prediabetes) | 15818 |
| 2 | Exercise (topic) OR Exercises (topic) OR Physical Activity (topic) OR Activities, Physical (topic) OR Activity, Physical (topic) OR Physical Activities (topic) OR Exercise, Physical (topic) OR Exercises, Physical (topic) OR Physical Exercise (topic) OR Physical Exercises (topic) OR Acute Exercise (topic) OR Acute Exercises (topic) OR Exercise, Acute (topic) OR Exercises, Acute (topic) OR Exercise, Isometric (topic) OR Exercises, Isometric (topic) OR Isometric Exercises (topic) OR Isometric Exercise (topic) OR Exercise, Aerobic (topic) OR Aerobic Exercise (topic) OR Aerobic Exercises (topic) OR Exercises, Aerobic (topic) OR Exercise Training (topic) OR Exercise Trainings (topic) OR Training, Exercise (topic) OR Trainings, Exercise (topic) | 1331299 |
| 3 | #2 AND #1 | 1955 |

Date: Sat Jan 07 2023 18:40:59 GMT+0800

| **Supplementary Table S2. Characteristics of included studies (n=10)** | | | | | | | | | | |
| --- | --- | --- | --- | --- | --- | --- | --- | --- | --- | --- |
| **Study** | **Region** | **Participants (AE/CG)** | | | | **Intervention** | | **Intensity** | **Frequency** | **Duration** |
|  |  | **N** | **Age (years)** | **Male (%)** | **Baseline BMI (kg/m²)** | **Aerobic exercise** | **Control** |  |  |  |
| Liu et al,  2020 | China | 43/43 | 60.35±4.29/  59.94±4.40 | 7/9 | 24.61±3.31/  24.60±2.04 | aerobic dancing and healthy diet | healthy diet | 60%-70% of HRmax | 3 times per week | 12 months |
| Yuan et al,  2019 | China | 83/83 | 60.93±5.71/  60.73±5.83 | 29/40 | 24.69±2.78/  25.04±2.86 | aerobic dancing and healthy diet | healthy diet | 60%-70% of HRmax | 3 times per week | 6 months |
| Yan et al,  2019 | China | 35/35 | 64.23±5.75/  60.31±7.56 | 29/43 | 22.37±3.44/  24.63±4.41 | aerobic dancing and healthy diet | healthy diet | 60%-70% of HRmax | 3 times per week | 12 months |
| Liu et al,  2013 | China | 20/21 | N/A | N/A | 27.10±1.28/  27.21±1.30 | walking exercise on a running machine | no intervention | 60%-70% of HRmax | 4 times per week | 6 months |
| Kim et al,  2022 | Korea | 15/9 | 70.47±5.57/  67.78±2.33 | N/A | 26.50±4.48/  24.86±2.23 | rhythmic physical activity | no intervention | 55%-65% of HRR/250-300kcal/day | 3 times per week | 3 months |
| Kargarfard et al, 2022 | Iran | 22/20 | N/A | 100/100 | 29.91±1.70/  29.72±1.26 | walking and jogging on  treadmill | no intervention | 50%-75% of HRRmax | 3 times per week | 3 months |
| He et al,  2022 | China | 56/55 | 60.93±5.71/  60.73±5.83 | 29/40 | 24.70 ±0.91/  25.04 ±0.92 | dancing with music and stretching exercises | no intervention | 60%-70% of HRmax | 3 times per week | 24 months |
| Dai et al,  2019 | China | 34/35 | N/A | N/A | N/A | aerobic dancing and weight maintenance diet | weight maintenance diet | 60%-70% of HRmax | 3 times per week | 24 months |
| Chen et al,  2021 | China | 83/83 | 60.93±5.71/  60.73±5.83 | 29/40 | 24.69±2.78/  25.04±2.86 | aerobic dancing and stretching exercises | no intervention | 60%-70% of HRmax | 3 times per week | 24 months |
| RezkAllah et al, 2019 | Egpyt | 20/20 | 31±5.27/  35.9±5.89 | 50/60 | 28.06±1.47/  28.41±1.37 | uphill running on a treadmill and low-calorie diet | low-calorie diet | HIIT up to 90% of HRmax | 3 times per week | 3 months |

Abbreviations: AE: aerobic exercise; BMI: body mass index; CG: control group; HRmax: maximal heart rate; HRR: heart rate reserve; HRRmax: maximum heart rate reserve; HIIT: high-intensity interval training; N/A: not available.
